# CRISPR-Cas9 engineered *Saccharomyces cerevisiae* for endolysin delivery to combat *Listeria monocytogenes*

**DOI:** 10.1007/s00253-025-13464-8

**Published:** 2025-04-02

**Authors:** David Sáez Moreno, Joana Cunha, Luís Daniel Rodrigues de Melo, Kenya Tanaka, Takahiro Bamba, Tomosiha Hasunuma, Joana Azeredo, Lucília Domingues

**Affiliations:** 1 CEB - Centre of Biological Engineering, University of Minho, Braga, Portugal; 2 LABBELS - Associate Laboratory, Braga, Guimarães, Portugal; 3 Faculty of Pharmacy, University of Coimbra, Coimbra, Portugal; 4 Engineering Biology Research Center, Kobe University, Nada, Kobe, Japan; 5 Graduate School of Science, Innovation and Technology, Kobe University, Nada, Kobe, Japan; 6 RIKEN Center for Sustainable Resource Science, 1-7-22 Suehiro-cho, Tsurumi-ku, Yokohama, Kanagawa 230-0045 Japan

**Keywords:** *Listeria monocytogenes*, Endolysin, CRISPR-Cas9, Biocontrol, Engineered *Saccharomyces cerevisiae*, Probiotics

## Abstract

**Abstract:**

Listeriosis is an infection caused by the consumption of food contaminated with *Listeria monocytogene*s. It leads to febrile gastroenteritis, central nervous system infections, and even death in risk populations. Bacteriophage endolysins selectively kill bacteria hydrolyzing their cell walls and have emerged as a potential tool for listeriosis control. Ply511 is an anti-*Listeria* endolysin that has activity against all serovars of *L. monocytogenes.* The yeast *Saccharomyces cerevisiae* has been used to produce endolysins for biocontrol, but prior efforts relied on plasmids, which can lead to gene loss and include selection markers unsuitable for therapeutic use. Integration of endolysins in its genome has also been previously demonstrated, relying however, on selection markers for selection and maintenance of the modifications. This study explores *S. cerevisiae* as a generally regarded as safe (GRAS) platform for producing and displaying Ply511 through CRISPR-Cas9 integration, offering a marker-free and stable solution for *Listeria* biocontrol. Our results demonstrate that the surface display of Ply511 does not lead to bacterial reduction. In contrast, we show that yeast secreting endolysin significantly reduces *L. monocytogenes* in cells, supernatants, and cell extracts*.* The strongest effect was observed with concentrated spent supernatant and cell extract, which reduced *L. monocytogenes* below the lower limit of quantification. Additionally, the spent supernatant exhibited active anti-*Listeria* activity in milk. This study highlights yeast-secreted endolysins as a promising platform for listeriosis control and demonstrates the yeast secretion of endolysins can be used for the biocontrol of pathogenic bacteria.

**Key points:**

• *S. cerevisiae was edited using CRISPR-Cas9 to display or secrete endolysin Ply511.*

• *Cells, supernatants, and extracts of yeast secreting Ply511 act against L. monocytogenes.*

• * Demonstrates the yeast-based delivery of endolysins to control L. monocytogenes.*

**Supplementary Information:**

The online version contains supplementary material available at 10.1007/s00253-025-13464-8.

## Introduction

*Listeria monocytogenes* is a Gram-positive pathogen causative of human and animal infections, normally acquired through ingestion of contaminated food. The range of foods linked to listeriosis goes from ready-to-eat products to fresh produce and dairy (Välimaa et al. 2015; Ricci et al. 2018; Ribeiro et al. 2023). Listeriosis can lead to severe systemic infections including meningitis, septicemia, and abortion in risk groups, with a high (20%) case-fatality rate (Ranjbar and Halaji 2018).

The food industry has established strict food safety protocols, such as pasteurization, refrigeration, and/or the use of preservatives. However, *L. monocytogenes* is able to survive in refrigerated temperatures and can be found in processing plants (Osek et al. 2022). As a result, there is a need to develop alternative strategies for control that can be applied in situ, both during food production and storage, to minimize contamination risk. There is a growing interest in the incorporation of yeast as probiotic supplement, especially in dairy products (Kazemi et al. 2025). Moreover, symptoms of listeriosis in humans are often treated with antibiotics (Hof et al. 1997; Ishihara and Akazawa 2023). However, antibiotic-resistant strains of *Listeria* have already been reported (Morvan et al. 2010), underscoring the urgent need for alternative treatments.

Bacteriophage endolysins, also known as enzybiotics, are enzymes that degrade bacterial peptidoglycan and have been proposed as an alternative to antibiotics to treat bacterial infections (Dams and Briers 2019). Endolysins are highly specific (Murray et al. 2021; Pottie et al. 2024), not harming beneficial microbes, which is a common concern for the food industry and for human health. Endolysin Ply511 has shown to be a promising tool for biocontrol since it is active against all serovars of *Listeria* spp. (Schmelcher et al. 2010; Eugster and Loessner 2012)*.* The use of endolysins against *Listeria* could serve as a treatment or as a way of prevention either in a food matrix, or in the human intestine, e.g., as probiotic or postbiotic, as it has been proposed before for endolysins (Pottie et al. 2024). These strategies, however, depend on the production and the delivery of an active endolysin against the pathogen of interest.

The yeast *Saccharomyces cerevisiae* is a generally recognized as safe (GRAS) organism and is widely used for the display and secretion of heterologous proteins. The use of *S. cerevisiae* for heterologous protein display on its surfaces has been shown to enhance protein stability (Chun et al. 2020), which can be beneficial in low pH environments, such as food matrices, or for gastrointestinal delivery. In fact, *S. cerevisiae* has previously been used to produce endolysins LysA and LysA2 against *Limosilactobacillus fermentum* and *Levilactobacillus brevis* (Khatibi et al. 2014) and to display endolysin LysKB317 against *L. fermentum* (Lu et al. 2023) or LysSA11 against *Staphylococcus aureus* (Chun et al. 2020). These studies showed that *S. cerevisiae* can be used for endolysin production; however, they rely on the use of plasmids (and their selection markers) or induction strategies, which would make application and commercialization challenging. In contrast, the use of CRISPR-Cas9 to integrate the genetic modification assures a marker-free, stable, and scarless chromosomal integration (Jessop-Fabre et al. 2016).

In this study, we report for the first time the use of CRISPR-Cas9 to engineer *S. cerevisiae* strains for the expression of the anti-*Listeria* endolysin Ply511 in both surface-displayed and secreted forms. We explore the potential of these engineered yeast strains as a platform for biocontrol applications, evaluating their activity against different serovars of *L. monocytogenes* and assessing their performance in a food matrix. This study lays the groundwork for the development of yeast-based systems for endolysin production, highlighting their potential in food safety and pathogen management strategies. By expressing endolysin, yeast can also be utilized as an innovative food additive, using its antimicrobial properties to target and lyse harmful bacteria, thereby enhancing food preservation and safety.

## Materials and methods

### *Escherichia coli* transformation and maintenance

*E. coli* DH5/NZY5α (Nzytech, Lisbon, Portugal) was used for plasmid construction and propagation. *E. coli* NZY5α transformation was conducted according to NZY5α competent cell protocol (Nzytech, Lisbon, Portugal). Briefly, 5 µL of assembled plasmids (2 to 20 ng/µL) was added to *E. coli* competent cells. After 30 min of incubation on ice, cells were heat shocked at 42 °C for 40 s. Then, cells were incubated on ice for 2 min and 150 µL of SOC medium (Nzytech, Lisbon, Portugal) was added. After growing for 1 h (37 °C, 200 revolutions per minute (rpm)), cells were plated on LB agar supplemented with ampicillin (100 µg/µL) and incubated overnight at 37 °C. Positive transformants were confirmed by colony PCR and were propagated. All the plasmids were purified using GenElute™ Plasmid Miniprep Kit (Sigma-Aldrich, Burlington, MA, USA). The correct cloning was verified using Sanger sequencing by Eurofins Genomics (Ebersberg, Germany). A list of primers used for colony PCR are provided in Supplemental Table S1.

### Plasmids

Table 1 lists all plasmids used in this study. Plasmids and primers utilized in cloning steps are shown in Supplemental Material (Supplemental Table S1). The In-Fusion HD Cloning Kit (Takara Bio, Shiga, Japan) was used for plasmid assembling. The integrative plasmid pCfB3035-Ply511-Sed1_SD, pCfB2904-Ply511-Sed1_SD, and pCfB2909-Ply511-Sed1_SD for cell surface display of endolysin Ply511 were constructed from the plasmid pCfB3035, pCfB2904, or pCfB2909 (Jessop-Fabre et al. 2016), for integration into *S. cerevisiae* chromosomes X-4, XI-3, and XII-5, respectively.

**Table 1:** Plasmids used in this study

Plasmid name	Description	Integration site	Reference
pCfB3035-Ply511-Sed1_SD	Plasmid containing DNA sequences for *Sed1* promoter, *Sed1*SS, *Ply511*, GS-based linker, *V5 epitope*, GS-based linker, HRV 3C cut site, *Sed1* anchor, *Sag*1 terminator, and homology arms for site X-4	X-4	This study
pCfB2904-Ply511-Sed1_SD	Plasmid containing DNA sequences for *Sed1* promoter, *Sed1*SS, *Ply511*, GS-based linker, *V5 epitope*, GS-based linker, HRV 3C cut site, *Sed1* anchor, *Sag*1 terminator, and homology arms for site XI-3	XI-3	This study
pCfB2909-Ply511-Sed1_SD	Plasmid containing DNA sequences for *Sed1* promoter, *Sed1*SS, *Ply511*, GS-based linker, *V5 epitope*, GS-based linker, HRV 3C cut site, *Sed1* anchor, *Sag*1 terminator, and homology arms for site XII-5	XII-5	This study
pCfB3035-Ply511_SEC	Plasmid containing DNA sequences for *Sed1* promoter, *Sed1*SS, *Ply511*, *Sag1* terminator, and homology arms for site X-4	X-4	This study
pCfB2904-Ply511_SEC	Plasmid containing DNA sequences for *Sed1* promoter, *Sed1*SS, *Ply511*, *Sag1* terminator, and homology arms for site XI-3	XI-3	This study
pCfB2909-Ply511_SEC	Plasmid containing DNA sequences for *Sed1* promoter, *Sed1*SS, *Ply511*, *Sag1* terminator, and homology arms for site XII-5	XII-5	This study
pCfB2312 (Cas9)	Cas9 expression plasmid for marker-free integration	-	Jessop-Fabre et al. (2016)
pCfB3042 (gRNA-X-4)	Guide RNA plasmid targeting site X-4	X-4	Jessop-Fabre et al. (2016)
pCfB3045 (gRNA-XI-3)	Guide RNA plasmid targeting site XI-3	XI-3	Jessop-Fabre et al. (2016)
pCfB3050 (gRNA-XII-5)	Guide RNA plasmid targeting site XII-5	XII-5	Jessop-Fabre et al. (2016)
pI2-EG-kanMX	Surface display cassette containing DNA sequences encoding *Sed1* promoter, *Sed1*SS, *Sed1* anchor, and *Sag1* terminator	-	Cunha et al. (2021)

Surface display cassette was amplified from pI2-EG-kanMX (Cunha et al. 2021) by substitution of *Trichoderma reesei* endoglucanase for the codon optimized cassette synthesized by GenScript (Rijswijk, Netherlands) containing DNA sequences for Ply511 (EC:3.5.1.28) gene for *S. cerevisiae* followed by a GS-based linker (GSSGGS)—epitope tag from *simian virus 5* (V5-tag)—GS-based linker (G_4_S)_3_ and the recognition and cleavage site for human rhinovirus 3C and PreScission proteases (HRV3C cut site).

The integrative plasmids pCfB3035-Ply511_SEC, pCfB2904-Ply511_SEC, and pCfB2909-Ply511_SEC were obtained from their surface display counterparts described above (Supplemental Table S1), using the In-Fusion HD Cloning Kit (Takara Bio, Shiga, Japan). Gene maps with annotated sequences are provided in Supplemental Figs. S1 and S2.

### Yeast transformation and maintenance

The yeast *S. cerevisiae* CEN.PK113-7D (Nijkamp et al. 2012) was used for transformation and is referred to as *S. cerevisiae* or yeast throughout the text.

*S. cerevisiae* was transformed using the PEG/lithium acetate method (Gietz and Schiestl 2007). To generate the engineered strains, first, yeast was transformed using a Cas9-expressing plasmid (pCf2312). Following that transformation, the guide RNA plasmid (pCFB3050, targeting to XII-5, X-4 or XI-3 integration site, respectively) was transformed into the Cas9-expressing strains together with the corresponding linearized integrative vector constructed. For multiple integrations, the yeast was simply curated from the plasmid carrying the guide RNA before the subsequent transformations targeting the other two integration sites. Before activity testing, all yeast were curated from both plasmids by serial passaging in liquid or agar without antibiotics.

Yeast strains were propagated at 30 °C and maintained at 4 °C on YPD plates (1% yeast extract, 2% peptone, 2% glucose, 2% agar). For plasmid-carrying yeast strains, YPD media was supplemented with 200 µg/mL of geneticin G418 (Cas9-expressing plasmid, pCf2312) or/and 100 µg/mL nourseothricin sulfate (guide RNA-containing plasmids).

For yeast colony PCR, a colony was picked with a sterile toothpick and transferred to a fresh plate. The rest of the colony was then diluted in 50 µL of NaOH 20 mM. After incubation at 95 °C for 5 min, 1 µL was added to a microtube containing NZYTaq II 2 × Green Master Mix (NZYtech, Lisbon, Portugal) and the primers to verify the correct transformations (Table 2).

**Table 2:** Primers used in this study for the verification of the chromosomal insertion

Primer ID	Sequence (5′–3′)	Target locus	Reference
905	CTCACAAAGGGACGAATCCT	X-4	Jessop-Fabre et al. (2016)
906	GACGGTACGTTGACCAGAG	X-4	Jessop-Fabre et al. (2016)
911	GTGCTTGATTTGCGTCATTC	XI-3	Jessop-Fabre et al. (2016)
912	CACATTGAGCGAATGAAACG	XI-3	Jessop-Fabre et al. (2016)
899	CCACCGAAGTTGATTTGCTT	XII-5	Jessop-Fabre et al. (2016)
900	GTGGGAGTAAGGGATCCTGT	XII-5	Jessop-Fabre et al. (2016)

### Fluorescence microscopy analysis

Yeast wild-type or *Scv-*Ply511-Sed1-XII-5 were cultured for 96 h at 30 °C and 200 rpm. After incubation, it was centrifuged for 5 min at 2000 g and washed three to five times with phosphate-buffered saline at pH 7.5 (PBS). The washed yeast suspension was added to 270 µL of a solution of PBS containing different concentrations of V5 Tag Monoclonal Antibody (TCM5) and PE-Cyanine7 (Thermo Fisher Scientific, Waltham, MA, USA) and incubated for 30 min at 4 °C in the dark.

After incubation, the labeled yeast cells were visualized using fluorescence microscopy Olympus (Tokyo, Japan) BX51 epifluorescence microscope with fluorescence illuminator, equipped with a U-RFL-T mercury lamp light source and fluorescence filter cubes. Imaging was performed using the filter cube with excitation 545–580 nm and emission 610 nm, which was selected as the closest match for PE-Cyanine7’s fluorescence properties. Exposure time varied from 3 to 5 s upon excitation. Bright-field images were taken as controls. Data was acquired with Olympus (Tokyo, Japan) cellSens soſtware.

### *Listeria monocytogenes* strains

*L. monocytogenes* strains used for this study were obtained from CECT (Valencia, Spain): CECT 911 (Serovar 1/2c), CECT 937 (Serovar 3b), CECT 938 (Serovar 3c), CECT 5672 (Serovar 4b), CECT 939 (Serovar 4c). Tryptic Soy Broth (Sigma-Aldrich, Burlington, MA, USA) was used for their growth. Tryptic Soy Broth with 1.5% agar or Oxford Selective Agar (Sigma-Aldrich, Burlington, MA, USA) was used for their maintenance and enumeration.

### Evaluation of enzymatic activity

To evaluate the enzymatic activity of yeast-expressed endolysin, we prepared a lawn of heat-killed *L. monocytogenes* on YPD-agar. *L. monocytogenes* strains were grown overnight in Tryptic Soy Broth (Sigma-Aldrich, Burlington, MA, USA) at 37 °C. The cell suspension was heat-killed by autoclaving at 121 °C for 20 min. The heat-killed bacterial cells were then concentrated 50–100 × by centrifugation and resuspended in YPD-agar (1.5% agar) to achieve an opaque, homogeneous mixture. Yeast were either spotted (5 µL) or spread (100 µL) onto the YPD-agar plates containing the heat-killed *L. monocytogenes* suspension. Control plates included spots of wild-type yeast (WT) and cell extract containing the endolysin alone as a positive control. Plates were incubated at 30 °C for 48 h. Peptidoglycan degradation was assessed by observing zones of clearing around yeast colonies or spots, indicating enzymatic activity.

To produce the cell extracts containing the endolysin Ply511, *E. coli BL21* (DE3) cells (ThermoFisher Scientific, Waltham, MA, USA), containing the recombinant plasmid (pQE-30_pHPL511 (Loessner et al. 1996)), were grown at 37 °C in LB medium supplemented with ampicillin at 100 mg/mL until an optical density at 600 of 0.5 − 0.6 was reached. Protein expression was induced with 1 mM isopropyl-β-thiogalactopyranoside (IPTG, Sigma-Aldrich, Burlington, MA, USA), followed by incubation for 24 h at 16 °C, 120 rpm. The disruption of cells was performed as described previously, through thaw-freezing cycles and sonication (Nogueira et al. 2021)*.* The crude cell extract resulting from the sonication was filtered and used as positive control.

### Evaluation of anti-Listeria activity of yeast cells

To evaluate the killing activity of yeast against *L. monocytogenes*, co-culture assays were conducted. Recombinant yeast cells were pre-inoculated in YPD broth overnight. Cells were then diluted to OD 0.1 in 20 mL of YPD, in a 100-mL Erlenmeyer flask and grown at 30 °C and 200 rpm for 96 h. Yeast cells were then centrifuged at 5000 × *g* for 10 min and washed three times with Tris buffer (50 mM Tris, 200 mM NaCl, pH 8.0) to a final concentration of ~ 10^9^ colony-forming units (CFU)/mL. *L. monocytogenes* cells were grown overnight, centrifuged at 9000 × *g* for 10 min, washed, and resuspended and diluted in the same Tris buffer, to a concentration of ~ 10^4^ CFU/mL.

Yeast and *L. monocytogenes* cells were mixed (900 µL with 100 µL, respectively) in a 24-well plate and incubated at 150 rpm and 30 °C. Co-cultures were sampled at 3 h, 6 h, and 24 h. Viable *L. monocytogenes* cells were quantified by plating serial dilutions onto Oxford Selective Agar (Sigma-Aldrich, Burlington, MA, USA), followed by incubation at 37 °C for 24 h or 48 h, and colony-forming units (CFU/mL) were calculated.

### Evaluation of anti-Listeria activity of yeast supernatants

To evaluate the killing activity of yeast against *L. monocytogenes*, co-culture assays were conducted. Recombinant yeast cells were pre-inoculated in YPD broth overnight. Cells were then diluted to OD 0.1 in 20 mL of YPD, in a 100-mL Erlenmeyer flask and grown at 30 °C and 200 rpm for 96 h. Yeast cells were then centrifuged at 5000* g* for 10 min and supernatants were recovered. This supernatant was then filtered through a 0.22-µm sterile polyethersulfone membrane filter to remove any residual yeast cells. The expected molecular weight of Ply511 is 36 kDa; therefore, to concentrate the protein, filtered supernatants were concentrated tenfold using Amicon Ultra-15 Centrifugal Filter Units with a 10-kDa molecular weight cut-off (MilliporeSigma, Burlington, MA, USA) by centrifugation at 5000 × *g* until the desired volume was reached. Concentrated supernatants were collected and stored at 4 °C if used on the same day, or at − 20 °C until further use.

*L. monocytogenes* cells were grown overnight, centrifuged at 9000 × *g* for 10 min, washed, and resuspended and diluted in Tris buffer (as mentioned above), to a concentration of ~ 10^4^ CFU/mL.

Concentrated supernatants (900 µL) and *L. monocytogenes* cells (100 µL) were mixed in a 24-well plate and incubated at 150 rpm and 30 °C. Co-cultures were sampled at 3, 6, and 24 h. Viable *L. monocytogenes* cells were quantified by plating serial dilutions onto Tryptic Soy Broth with 1.5% agar (Sigma-Aldrich, Burlington, MA, USA), followed by incubation at 37 °C for 24 h, and colony-forming units (CFU/mL) were calculated.

For the evaluation of the anti-Listeria activity in milk, concentrated supernatants (400 µL) and *L. monocytogenes* cells diluted in semi-skimmed milk (50 µL) were mixed with semi-skimmed milk (350 µL) in a 24-well plate and incubated at 150 rpm and 4 °C. Semi-skimmed milk was previously pasteurized by heating the product to 90 °C for 30 min. Samples were taken at 6, 24, and 48 h. Viable *L. monocytogenes* cells were quantified by plating serial dilutions onto Oxford Selective Agar (Sigma-Aldrich, Burlington, MA, USA), followed by incubation at 37 °C for 24 h or 48 h, and colony-forming units (CFU/mL) were calculated.

### Evaluation of anti-Listeria activity of yeast extracts

To evaluate the killing activity of yeast against *L. monocytogenes*, co-culture assays were conducted. Recombinant yeast cells were pre-inoculated in YPD broth overnight. Cells were then diluted to OD 0.1 in 20 mL of YPD, in a 100-mL Erlenmeyer flask and grown at 30 °C and 200 rpm for 96 h. Yeast cells were then centrifuged at 5000 g for 10 min and washed with Tris buffer (50 mM Tris, 200 mM NaCl, pH 8.0) to a final concentration of ~ 10^9^ CFU/mL.

To prepare the sonicated yeast extract, cell suspensions were subjected to sonication using a Cole‐Parmer Ultrasonic Processor (Cole-Parmer, Vernon Hills, IL, USA). Sonication was carried out in three cycles of 5 min each, with 10 intervals of 30 s ON and 30 s OFF at 40% amplitude. The sonicated extract was then centrifuged at 10,000 × *g* for 10 min to remove cell debris, and the supernatant was filtered through a 0.22-µm sterile filter (Millipore-Sigma, Burlington, MA, USA) to ensure sterility. The filtered sonicated extract was stored at 4 °C if used on the same day, or at − 20 °C until further use.

*L. monocytogenes* cells were grown overnight, centrifuged at 9000* g* for 10 min, washed, and resuspended and diluted in the same Tris buffer, to a concentration of ~ 10^4^ CFU/ml.

Yeast extracts (900 µL) and *L. monocytogenes* cells (100 µL) were mixed in a 24-well plate and incubated at 150 rpm and 30 °C. Co-cultures were sampled at 3, 6, and 24 h. Viable *L. monocytogenes* cells were quantified by plating serial dilutions onto Tryptic Soy Broth with 1.5% agar (Sigma-Aldrich, Burlington, MA, USA), followed by incubation at 37 °C for 24 h, and colony-forming units (CFU/mL) were calculated.

### Yeast growth monitoring

To assess yeast growth, cultures were monitored in a 96-well plate using an automated system for real-time biomass evaluation. Overnight cultures were initially grown in YPD broth at 30 °C and subsequently diluted 1:100 in fresh YPD to a final working volume of 200 µL per well. Each well was inoculated with a standardized yeast suspension, and plates were incubated in a BioLector XT microbioreactor (Beckman Coulter, Brea, CA, USA) at 30 °C with shaking at 800 rpm. Optical density (OD) measurements (biomass) were recorded every 15 min to capture growth kinetics over time.

Yeast growth was also quantified by plating serial dilutions on YPD agar. Samples from the 96-well plate were serially diluted in saline (0.9% NaCl solution) and plated onto YPD agar to assess colony-forming units which were counted and photographed.

### Statistics

All data analysis was performed in GraphPad (version 9.0.0). Unpaired *t*-test were used to evaluate statistical significance. The upper threshold for statistical significance for all experiments was set at *p* < 0.05.

## Results

### Construction of various novel Ply511-displaying yeast strains

**Fig. 1:**
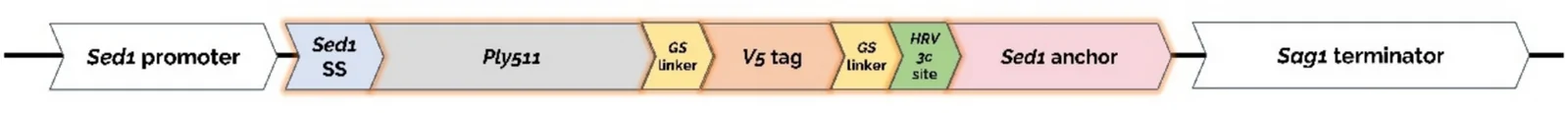
Endolysin display cassette integrated into *S. cerevisiae*. The coding sequence is highlighted with orange (*Sed1* secretion signal (*Sed1*SS)-*Ply511*-GS-based linker (GSSGGS)-*V5*tag-GS-based linker(G_4_S)_3_-*HRV3C* cut site-*Sed1* anchor protein), preceded by the *Sed1* promoter and followed by the *Sag1* terminator. *V5*tag refers to epitope tag from *simian virus 5*

To test the yeast surface display of the endolysin Ply511, i.e., a fusion protein between a protein of the yeast cell wall (Sed1) and a protein of interest (endolysin Ply511), *S. cerevisiae* CEN.PK113-7D was used. Using CRISPR-Cas 9, the endolysin-expressing cassette was integrated in one, two, or three different loci (chromosomes X, XI, and XII, Table 3), previously reported to lead to stable insertions (Jessop-Fabre et al. 2016). The endolysin expression cassette included the sequences of the *Sed1* promoter, for the Sed1 secretion signal peptide (Sed1SS), endolysin Ply511 fused with a flexible linker, a V5 epitope tag, an additional GS-based linker, the Sed1 anchor protein, and the *Sag1* terminator (Fig. 1). The choice of promoter, anchor, and terminator pair was done according to previous optimization (Inokuma et al. 2016).

**Table 3:** Yeast strains constructed in this study from *S. cerevisiae* CEN.PK113-7D

Strain designation	Modified locus	Donor plasmid features	Number of copies
*Scv-*Ply511-Sed1-XII-5	XII-5	Sequences encoding for *Sed1* promoter, Sed1 secretion signal, Ply511, GS-based linker (GSSGGS)-, V5 epitope, GS-based linker(G_4_S)_3_, HRV 3C cut site, Sed1 anchor, *Sag1* terminator	1
*Scv-*Ply511-Sed1-XII-5-XI-3	XII-5; XI-3	Same as above	2
*Scv-*Ply511-Sed1-X-4-XI-3-XII-5	X-4; XI-3; XII-5	Same as above	3

The constructs were integrated in their respective locus and the correct integration was confirmed by diagnosis PCR (Supplemental Fig. S3). The strains constructed are listed in Table 3.

We observed no difference in growth between the wild-type (WT) strain and the constructed strains, except for the strain with the triple integration (*Scv*-Ply511-Sed1-X-4-XI-3-XII-5), which showed significantly impaired growth over a 24-h period, as shown in Supplemental Fig. S4.

### V5 epitope can be detected and used as indicator of protein expression in the yeast surface

**Fig. 2:**
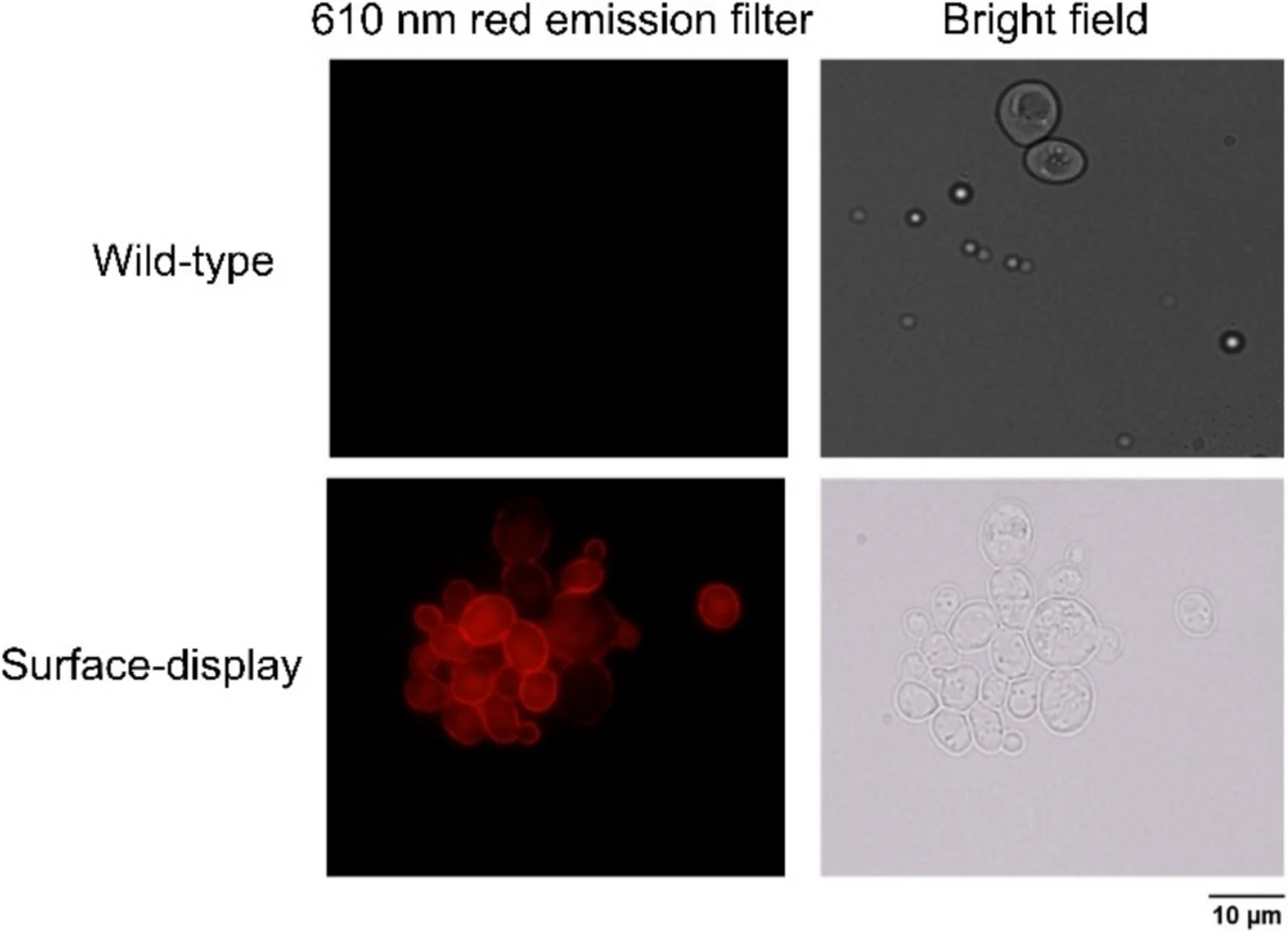
Microscopic images of wild-type yeast and yeast displaying Ply511 tagged with an anti-V5 antibody, captured under bright field and a 610-nm red emission filter, as indicated in the legend

To confirm the efficient display of the Ply511 endolysin fused to the Sed1 anchor protein, a V5 epitope tag was added to the linker (Fig. 1). Using an anti-V5 tag fluorescent antibody, the labelled cells were visualized using fluorescent microscopy, showing the display of the protein of interest uniformly on the cell-wall of the yeast (Fig. 2).

### *S. cerevisiae* displaying endolysin Ply511 shows enzymatic activity

**Fig. 3:**
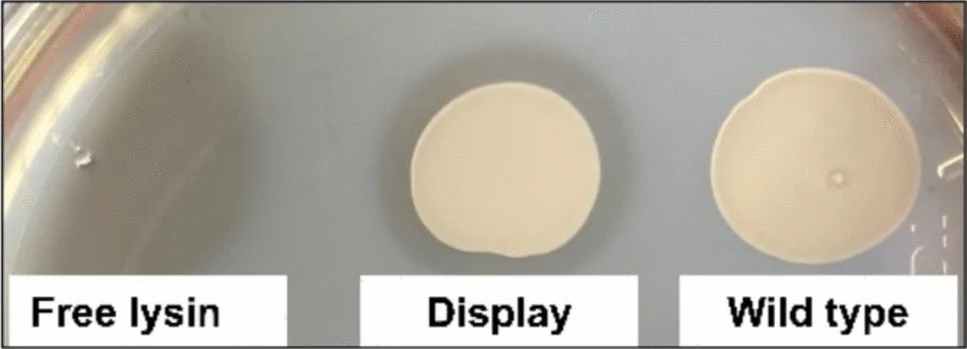
Enzymatic activity of *S. cerevisiae* displaying endolysin Ply511. Effect of the free endolysin Ply511, the yeast displaying Ply511, and the wild-type yeast over a heat-killed layer of *L. monocytogenes* CECT 939, serovar 4c

To confirm the enzymatic activity of the endolysin, we tested the yeast’s ability to degrade the peptidoglycan in a lawn of heat-killed bacteria*.* Recombinant yeast was spotted or spread over an opaque YPD-agar mixture containing heat-killed *L. monocytogenes.* We observed that after 48 h of incubation, the recombinant yeast cleared the opaque surface around its growth by degrading the *L. monocytogenes* peptidoglycan layer (Fig. 3), similar to the activity of free endolysin and in contrast to WT yeast (Fig. 3). This activity was consistent across all *Listeria* spp. serovars tested (Supplemental Table S2), indicating that the enzyme, when fused to the yeast cell wall, retains its peptidoglycan degradation function*.*

### *S. cerevisiae* displaying endolysin Ply511 does not have antimicrobial activity and its growth is impaired

To assess whether the recombinant yeast could effectively reduce *L. monocytogenes* in co-culture, we mixed the two species in buffer (Tris Buffer 50 mM, 200 mM NaCl at pH 8.0) and monitored viable *L. monocytogenes* cells over time. As shown in Supplemental Table S3, no significant difference in *L. monocytogenes* log_10_ (CFU/mL) was observed at 3, 6, or 24 h, even at varying yeast concentrations. Interestingly, *L. monocytogenes* continued to grow in the presence of yeast over the 24-h period. This lack of killing activity was consistent regardless of the cassette copy number (1, 2, or 3) integrated into the yeast chromosome, the buffer type (20 mM Citrate, pH 5.0; Tris, pH 8.0; or PBS, pH 7.4), the *L. monocytogenes* growth phase (exponential or stationary), or the anchor protein used (Sed1 or Sag1) (data not shown).

### *S. cerevisiae* triple integration of the Ply511 secretion cassette does not affect yeast growth

**Fig. 4:**
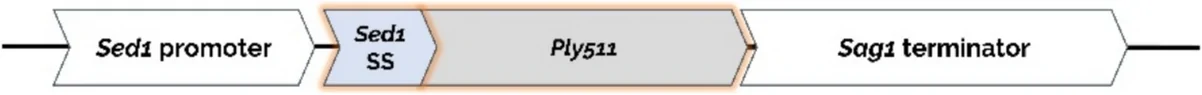
Endolysin secretion cassette integrated into *S. cerevisiae*. The coding sequence is highlighted with orange (*Sed1* secretion signal-*Ply511*), preceded by the *Sed1* promoter and followed by the *Sag1* terminator

We hypothesized that the growth impairment of the yeast carrying three copies of the display of Ply511 could be due to the over-expression and saturation of the Sed1 protein in the yeast surface. Additionally, the lack of killing activity might be due to the endolysin being immobilized on the yeast cell wall, which may prevent it from freely accessing and reaching the bacterial peptidoglycan. To enhance *S. cerevisiae*’s anti-*Listeria* activity, we removed the sequences for the *Sed1* anchor from our construct, designing it to secrete Ply511 into the extracellular medium. The secretion cassette included the sequences for the *Sed1* promoter, the *Sed1* secretion signal peptide, the *Ply511* endolysin, and the *Sag1* terminator (Fig. 4). Using CRISPR-Cas9, we integrated the endolysin-expressing cassette into three loci on chromosomes X, XI, and XII (Supplemental Fig. S3) creating the strain *Scv-*Ply511-X-4-XI-3-XII-5.

Unlike the yeast with triple integration for surface display, the yeast secreting endolysin showed no growth impairment, achieving a biomass growth rate similar to the WT in both broth and on plate (Supplemental Fig. S5a and b).

### *S. cerevisiae* secreting Ply511 is active against* L. monocytogenes* in co-culture

**Fig. 5:**
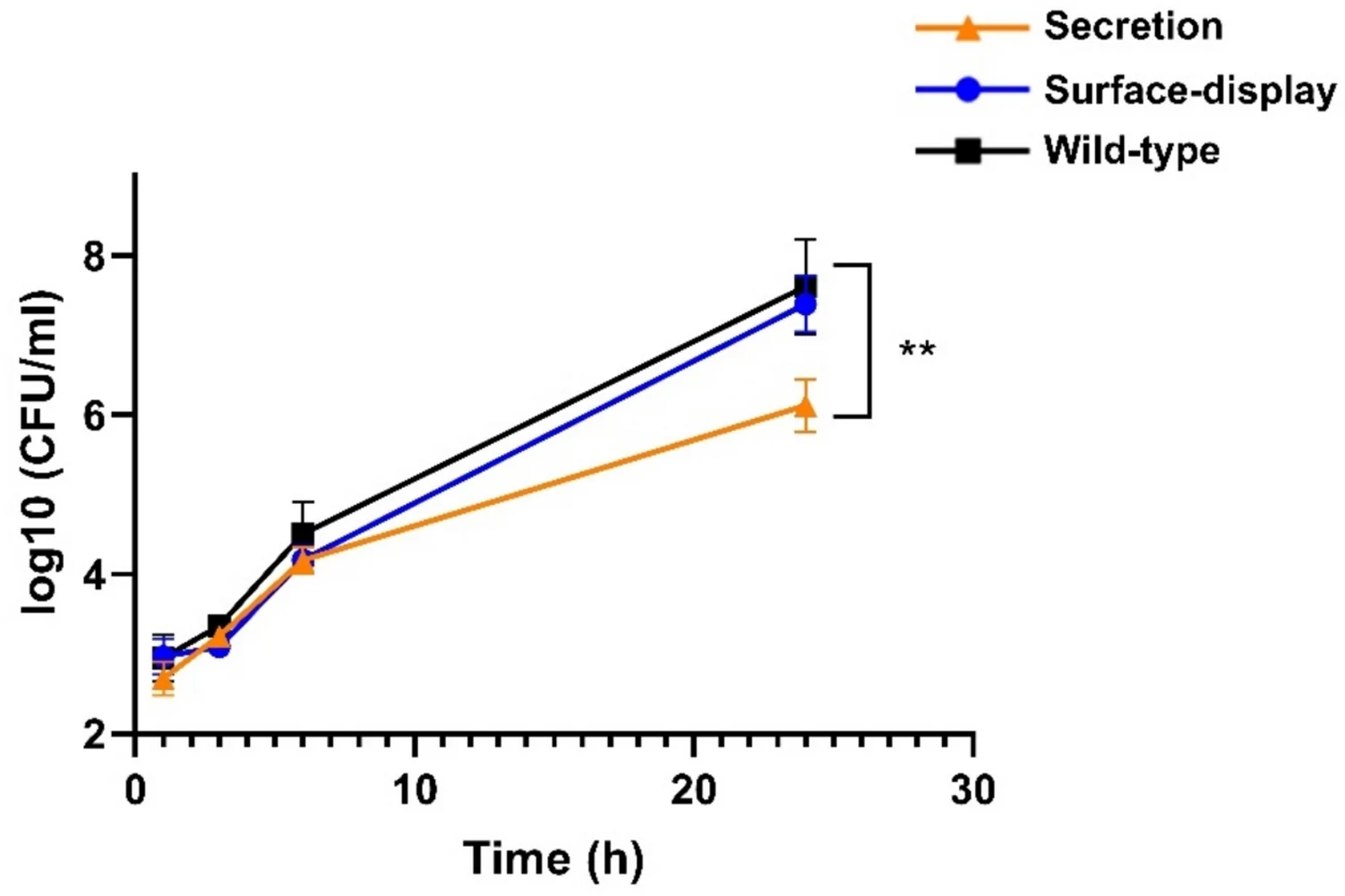
Log_10_ (CFU/mL) of *L. monocytogenes* serovar 4c upon mixture with *S. cerevisiae* as indicated in the legend. The figure shows the log_10_ (CFU/mL) mean of three replicates; standard deviation is represented as error bars. The unpaired *t* test comparison between the WT yeast and the yeast secreting Ply511 at 24 h is indicated as follows: ****p ≤ 0.0001, ***p ≤ 0.001, **p ≤ 0.01, and *p ≤ 0.05

To assess if the recombinant yeast could effectively reduce *L. monocytogenes* serovar 4c in co-culture, we mixed bacterial and yeast cells in Tris Buffer and monitored viable *L. monocytogenes* cells over time. As shown in Fig. 5, the recombinant yeast secreting endolysin significantly reduced *L. monocytogenes* levels after 24 h of contact.

### Spent supernatant from *S. cerevisiae* secreting Ply511 shows anti-Listeria killing activity

**Fig. 6:**
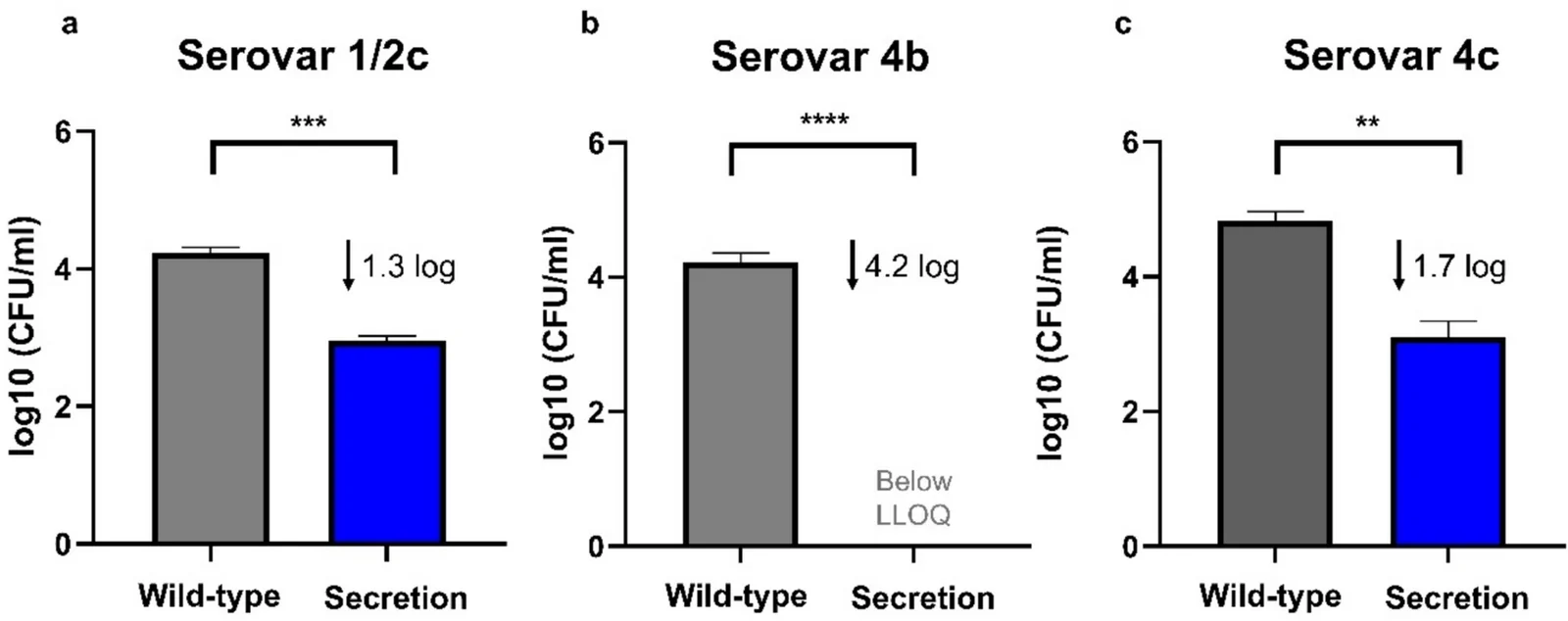
Log_10_ (CFU/mL) of *L. monocytogenes* upon mixture with *S. cerevisiae* spent supernatant concentrated (10 ×) from WT or SEC (secreting Ply511) after a period of 6 h. **a** serovar 1/2c, **b** serovar 4b, and **c** serovar 4c. The figure shows the log_10_ (CFU/mL) mean of three replicates; standard deviation is represented as error bars and the unpaired *t* test comparison between the two groups, indicated as follows: ***p ≤ 0.0001, ***p ≤ 0.001, **p ≤ 0.01, and *p ≤ 0.05. The lower limit of quantification (LLOQ) in this assay was 2.69 log10 (CFU/mL)

To confirm that the yeast was effectively secreting an active protein, we tested the antimicrobial activity of the spent supernatants against three different *L. monocytogenes* serovars (1/2c, 4b, and 4c). As shown in Fig. 6a, after 6 h, the concentrated (10 ×) supernatant reduced 1.3 log_10_ (CFU/mL) in serovar 1/2c (Fig. 6a), 4.2 log_10_ (CFU/mL) in serovar 4b (Fig. 6b), and 1.7 log_10_ (CFU/mL) in serovar 4c (Fig. 6c), compared to the concentrated supernatant from the WT yeast.

### Spent supernatant shows anti-Listeria killing activity in milk

**Fig. 7:**
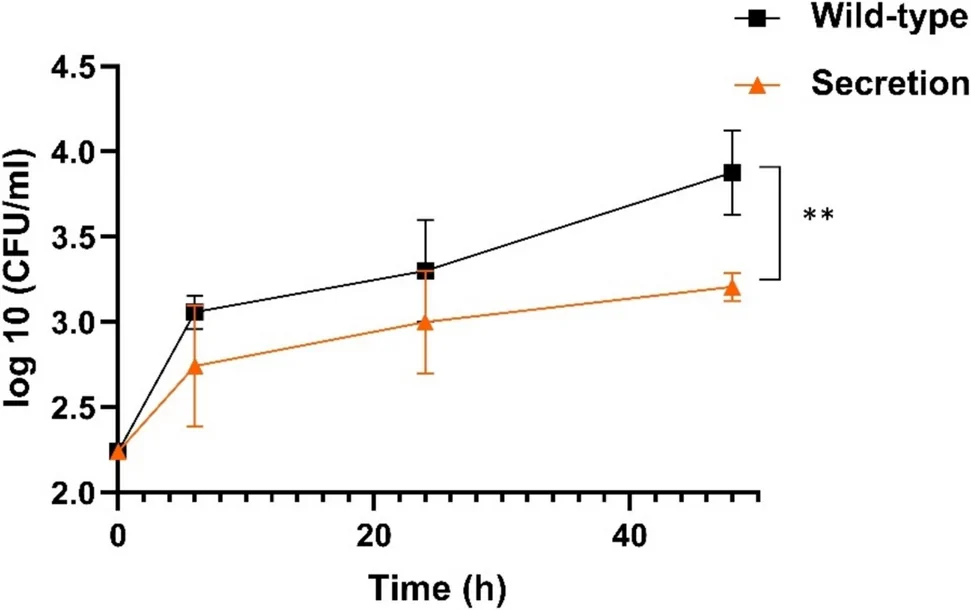
Activity of supernatants in milk. The figure represents the time-course (6 h, 24 h and 48 h of log_10_ (CFU/mL) of *L. monocytogenes* Serovar 4b upon mixture with *S. cerevisiae* cell extract from WT or SEC (secreting Ply511). The figure shows the log_10_ (CFU/mL) mean of three replicates and standard deviation is represented as error bars. The unpaired t test comparison between the two groups at 48 h, is indicated as follows: ****p ≤ 0.0001, ***p ≤ 0.001, **p ≤ 0.01, and *p ≤ 0.05

After confirming the activity of the supernatants, we investigated whether they could effectively reduce *L. monocytogenes* in milk—a food product highly susceptible to contamination with this bacterium. We mixed *L. monocytogenes* serovar 4b with 10 × concentrated spent supernatant at 4 °C in a 1:1 supernatant-to-milk ratio. As shown in Fig. 7, both mixtures allowed *L. monocytogenes* to grow over 48 h, which may result from the nutrients left in the spent yeast supernatants or from the ability of *L. monocytogenes* to grow in milk. However, at 6, 24, and 48 h, the supernatant from yeast secreting Ply511 reduced *L. monocytogenes* CFU/mL by 40%, 50%, and 80%, respectively, compared to WT supernatant. Only the reduction at 48 h was statistically significant, corresponding to a decrease of 0.7 log_10_ (CFU/mL).

### Improved anti-Listeria killing activity in yeast cell extracts

**Fig. 8:**
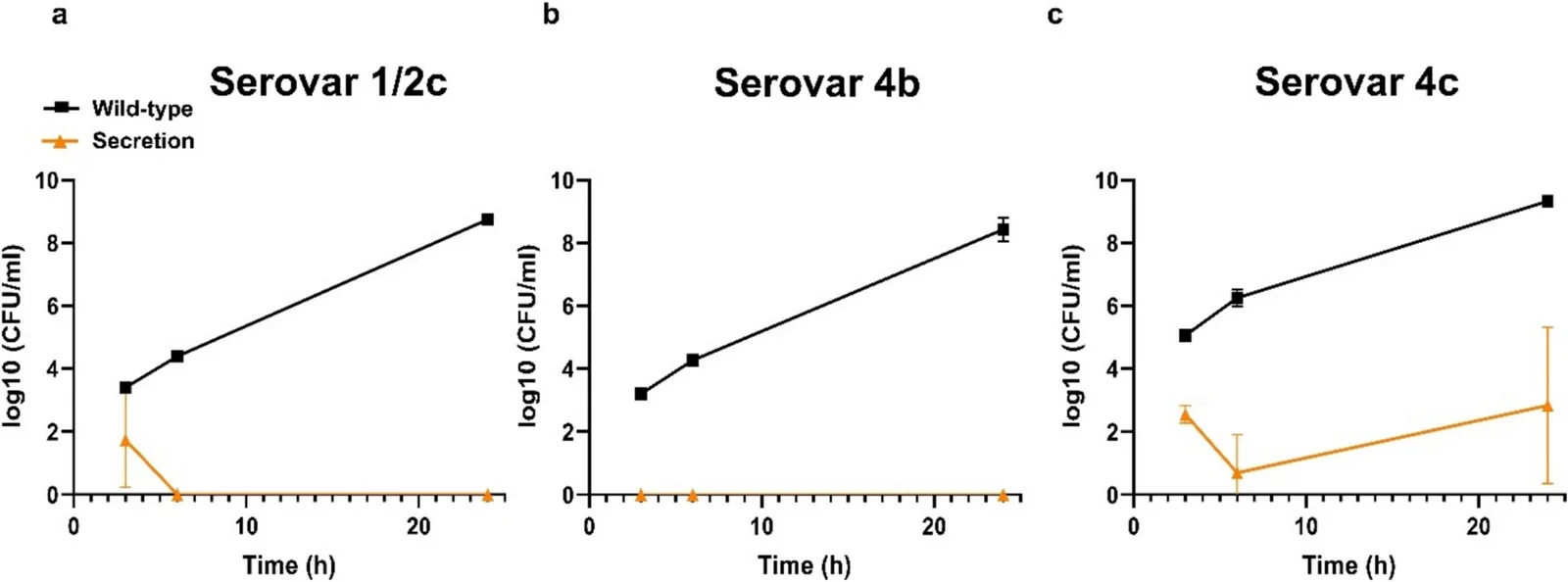
Time-course of log10 (CFU/mL) of *L. monocytogenes* upon mixture with *S. cerevisiae* cell extract from WT or SEC (secreting Ply511). **a** Serovar 1/2c, **b** Serovar 4b, and **c** Serovar 4c. Values represent the mean of three replicates and standard deviation is represented as error bars. The lower limit of quantification in this assay was 2.69 log_10_ (CFU/mL). SEC stands for yeast secreting Ply511 and WT stands for wild-type yeast

Since both yeast cells and supernatant were found to be active individually, we hypothesized that the yeast fraction might still contain some protein. To test this, we mechanically lysed the yeast cells and recovered the soluble fraction, referred to as the yeast extract. When tested against *L. monocytogenes*, we observed that while the WT yeast extract supported *L. monocytogenes* growth, the extract from engineered yeast significantly reduced *L. monocytogenes* cells to below the lower limit of quantification for serovars 1/2c (Fig. 8a) and 4b (Fig. 8b). Specifically, WT yeast extract supported growth to 8.74 and 8.42 log_10_ (CFU/mL) for serovars 1/2c and 4b, respectively, after 24 h. In contrast, no viable cells were recovered at 6 or 24 h with the engineered yeast extract. Against serovar 4c, the engineered yeast extract reduced *L. monocytogenes* by an average of 6.5 log_10_ (CFU/mL) after 24 h (Fig. 8c).

## Discussion

In this study, we analyzed the capacity of *S. cerevisiae* to display or secrete the anti-*Listeria* endolysin Ply511, demonstrating the potential of a yeast-based platform for endolysins expression to control listeriosis, with potential applications in food biopreservation and pathogen biocontrol.

First, we demonstrate that our engineered yeast strain, which features triple integration of the expression cassette at three different chromosomal sites, results in the production of an enzymatically active protein through both display and secretion. Since listeriosis is frequently acquired through contaminated food, this modified yeast has the potential to contribute to in situ bio-preservation of certain food products. For applications in humans, food, or feed, the genetic engineering strategy must avoid antibiotic resistance markers and ensure the stability of the modification for effective protein delivery. CRISPR-Cas9 allows for multiple integrations, enables scarless insertion, and provides greater stability compared to plasmid-driven expression, while also eliminating the need for selection markers (Jessop-Fabre et al. 2016). This approach not only reduces production costs but may also offer regulatory advantages for applications in both food safety and human therapy.

Surprisingly, we found that displaying the endolysin Ply511 on the surface of *S. cerevisiae* did not inhibit the growth of *L. monocytogenes* compared with the WT strain. These negative results led us to conclude that the activity of the displayed endolysin is highly protein or host dependent. For instance, Chun et al. (2020) reported strong activity for the endolysin LysSA11 against *S. aureus*, while Lu et al. (2023) demonstrated the activity of yeast surface display of LysKB317 against *Limosilactobacillus fermentum.* The lack of activity observed in our study may stem from the endolysin’s mode of action, which requires diffusion through the bacterial peptidoglycan to access its substrate. This diffusion is significantly hindered when the protein is anchored to the yeast cell wall and might not be enough to achieve *L. monocytogenes* killing. Our results suggest that this effect is protein or host dependent; however, both Chun et al. (2020) and Lu et al. (2023) used a different yeast host (EBY100) and a different anchor protein for the display (Aga2p) of both factors that influence protein activity. Sed1 and Sag1 anchors allow only for an N-terminal fused anchor; we tested this configuration since Lu et al. (2023) showed that N- or C-terminal configuration did not influence activity. However, this was not tested for Ply511 endolysin. Also, further increasing linker length to avoid steric hindrance may have a positive influence in activity (Tanaka and Kondo 2015; Cunha et al. 2021).

To circumvent the lack of activity from the surface display, we decided to use the yeast for secreting the endolysin. Our results show that both the yeast cells and their spent supernatant effectively reduced *L. monocytogenes* levels. Furthermore, we improved this activity by subjecting the yeast cells to a simple mechanical lysis process to obtain a yeast extract. This approach offers an interesting application of this technology, allowing the yeast to deliver the protein in food matrices or within the gut, acting as a probiotic, or to release the protein at the site of interest without the need to keep cells alive, thereby acting as postbiotic. Such strategies may be particularly relevant for controlling *L. monocytogenes*, a foodborne pathogen that first interacts with the gut before potentially entering the bloodstream (Barbuddhe and Chakraborty 2009).

We observed that the Sed1 secretion signal peptide could effectively drive the secretion of Ply511 into the extracellular media, as previously shown with other proteins (Inokuma et al. 2016). However, we also found that yeast cells, even after extensive wash and without supernatant, were still capable of reducing *L. monocytogenes* levels*.* This suggests that the secretion signal may be directing some of the protein to the cell membrane. We have previously observed this phenomenon with other proteins, further supporting this hypothesis (data not shown) and with a different secretion signal (Matano et al. 2013). The fact that yeast cells reduce *L. monocytogenes* after 24hs but not at earlier time-points, as shown in Fig. 5, suggests that yeast might have some metabolic activity after several hours in buffer, thus releasing active protein and/or that there is some yeast cell lysis, which might cause the release of protein. This causes the activity not to be immediate, contrary to what would happen with purified endolysins.

The endolysin Ply511 was previously expressed in four different lactic acid bacteria (Turner et al. 2007); however, none of its supernatants could control *L. monocyogenes* growth. Our study represents the first report where Ply511 has been effectively secreted in an active form and showing killing activity. The activity of the endolysin produced in yeast supernatants might be different from that of the protein being produced in *E. coli*, due to yeast ability to glycosylate proteins. Although the glycosylation of Ply511 was not directly verified in this study, the observed activity of yeast supernatants suggests that the potential glycosylation involved in the secretion does not abolish the activity of this endolysin.

Strategies such as promoter engineering, secretion signal engineering, genetic modifications of native genes involved in the secretory pathway, or protease knockouts have been shown to improve *S. cerevisiae* secretion of heterologous proteins (Yang et al. 2024). These strategies and better understanding of the *Sed1*SS-driven secretory pathway would allow to further improve the secretion of the endolysin, as evidenced by the fact that some protein is retained inside of the cells, ultimately leading to a higher anti-*Listeria* activity.

The engineered yeast supernatants and extracts demonstrate efficacy against *L. monocytogenes* serovars 1/2c, 4b, and 4c, underscoring the broad host range of this endolysin (Schmelcher et al. 2010; Eugster and Loessner 2012). Serovar 4b exhibited the highest susceptibility to Ply511, which is significant given its prevalence in foodborne outbreaks (Gray et al. 2004; Amato et al. 2017; Ferreira et al. 2018). Moreover, serovar 4b is linked to a high fatality rate and is commonly found in dairy products (Amato et al. 2017; Ferreira et al. 2018). Thus, the activity of these spent supernatants in milk is particularly promising for the potential application of this modified yeast in food preservation.

This study demonstrates the innovative delivery of phage endolysins via yeast, showing the potential of this technology to effectively combat *L. monocytogenes*. Future in vivo tests and assessment of susceptibility to digestion are crucial next steps in advancing this promising application. The vision for this yeast-based platform extends beyond just *L. monocytogenes* and holds significant potential targeting a variety of gut-related pathogens offering a versatile and effective approach to enhancing food safety and public health.

## Supplementary Information

Below is the link to the electronic supplementary material.

Supplementary file1 (PDF 670 kb)

## Data Availability

The data presented in this study are available within the article and supplementary material.

## Declarations

This article contained no studies incorporating human participants or animals to be performed by any of the authors.

## References

[CR1] Amato E, Filipello V, Gori M, Lomonaco S, Losio MN, Parisi A, Huedo P, Knabel SJ, Pontello M (2017) Identification of a major *Listeria **monocytogenes* outbreak clone linked to soft cheese in Northern Italy - 2009–2011. BMC Infect Dis 17:1–7. 10.1186/S12879-017-2441-6 28499357 10.1186/s12879-017-2441-6 PMC5429568

[CR2] Barbuddhe SB, Chakraborty T (2009) *Listeria* as an enteroinvasive gastrointestinal pathogen. Curr Top Microbiol Immunol 337:173–195. 10.1007/978-3-642-01846-6_6 19812983 10.1007/978-3-642-01846-6_6

[CR3] Chun J, Bai J, Ryu S (2020) Yeast surface display system for facilitated production and application of phage endolysin. ACS Synth Biol 9:508–516. 10.1021/ACSSYNBIO.9B00360 32119773 10.1021/acssynbio.9b00360

[CR4] Cunha JT, Gomes DG, Romaní A, Inokuma K, Hasunuma T, Kondo A, Domingues L (2021) Cell surface engineering of *Saccharomyces **cerevisiae* for simultaneous valorization of corn cob and cheese whey via ethanol production. Energy Convers Manag 243:114359. 10.1016/j.enconman.2021.114359

[CR5] Dams D, Briers Y (2019) Enzybiotics: enzyme-based antibacterials as therapeutics. Adv Exp Med Biol 1148:233–253. 10.1007/978-981-13-7709-9_11 31482502 10.1007/978-981-13-7709-9_11

[CR6] Eugster MR, Loessner MJ (2012) Wall teichoic acids restrict access of bacteriophage endolysin Ply118, Ply511, and PlyP40 cell wall binding domains to the *Listeria **monocytogenes* peptidoglycan. J Bacteriol 194:6498. 10.1128/JB.00808-12 23002226 10.1128/JB.00808-12 PMC3497465

[CR7] Ferreira V, Magalhães R, Almeida G, Cabanes D, Fritzenwanker M, Chakraborty T, Hain T, Teixeira P (2018) Genome sequence of *Listeria **monocytogenes* 2542, a serotype 4b strain from a cheese-related outbreak in Portugal. Genome Announc 6:e00540-e618. 10.1128/GENOMEA.00540-18 29930053 10.1128/genomeA.00540-18 PMC6013623

[CR8] Gietz RD, Schiestl RH (2007) High-efficiency yeast transformation using the LiAc/SS carrier DNA/PEG method. Nat Protoc 2:31–34. 10.1038/nprot.2007.13 17401334 10.1038/nprot.2007.13

[CR9] Gray MJ, Zadoks RN, Fortes ED, Dogan B, Cai S, Chen Y, Scott VN, Gombas DE, Boor KJ, Wiedmann M (2004) *Listeria **monocytogenes* isolates from foods and humans form distinct but overlapping populations. Appl Environ Microb 70:5833–5841. 10.1128/AEM.70.10.5833-5841.2004/10.1128/AEM.70.10.5833-5841.2004 PMC522108 15466521

[CR10] Hof H, Nichterlein T, Kretschmar M (1997) Management of listeriosis. Clin Microbiol Rev 10:345–357. 10.1128/CMR.10.2.345 9105758 10.1128/cmr.10.2.345 PMC172923

[CR11] Inokuma K, Hasunuma T, Kondo A (2014) Efficient yeast cell-surface display of exo- and endo-cellulase using the SED1 anchoring region and its original promoter. Biotech Biof 7:8. 10.1186/1754-6834-7-8 10.1186/1754-6834-7-8 PMC3900695 24423072

[CR12] Inokuma K, Bamba T, Ishii J, Ito Y, Hasunuma T, Kondo A (2016) Enhanced cell-surface display and secretory production of cellulolytic enzymes with *Saccharomyces **cerevisiae* Sed1 signal peptide. Biotech Bioeng 113:2358–2366. 10.1002/BIT.26008 10.1002/bit.26008 27183011

[CR13] Ishihara Y, Akazawa K (2023) Treatment of *Listeria **monocytogenes* bacteremia with oral levofloxacin in an immunocompromised patient. Idcases 31:e01680. 10.1016/J.IDCR.2023.E01680 36660737 10.1016/j.idcr.2023.e01680 PMC9843167

[CR14] Jessop-Fabre MM, Jakočiūnas T, Stovicek V, Dai Z, Jensen MK, Keasling JD, Borodina I (2016) EasyClone-MarkerFree: a vector toolkit for marker-less integration of genes into *Saccharomyces **cerevisiae* via CRISPR-Cas9. Biotechnol J 11:1110–1117. 10.1002/BIOT.201600147 27166612 10.1002/biot.201600147 PMC5094547

[CR15] Kazemi S, Homayouni-Rad A, SamadiKafil H, Sarabi-Aghdam V, Zeynolabedini P, Pour Agha B, Allah Madadi S (2025) Selection of appropriate probiotic yeasts for use in dairy products: a narrative review. Food Prod Proc Nutr 7:13. 10.1186/S43014-024-00293-X

[CR16] Khatibi PA, Roach DR, Donovan DM, Hughes SR, Bischoff KM (2014) *Saccharomyces **cerevisiae* expressing bacteriophage endolysins reduce *Lactobacillus* contamination during fermentation. Biotech Biol 7:104. 10.1186/1754-6834-7-104/

[CR17] Loessner MJ, Schneider A, Scherer S (1996) Modified *Listeria* bacteriophage lysin genes (ply) allow efficient overexpression and one-step purification of biochemically active fusion proteins. Appl Environ Microb 62:3057–3060. 10.1128/AEM.62.8.3057-3060.1996 10.1128/aem.62.8.3057-3060.1996 PMC168095 8702301

[CR18] Lu SY, Liu S, Patel MH, Glenzinski KM, Skory CD (2023) *Saccharomyces **cerevisiae* surface display of endolysin LysKB317 for control of bacterial contamination in corn ethanol fermentations. Front Bioeng Biotechnol 11:1162720. 10.3389/FBIOE.2023.1162720 37091344 10.3389/fbioe.2023.1162720 PMC10117863

[CR19] Matano Y, Hasunuma T, Kondo A (2013) Cell recycle batch fermentation of high-solid lignocellulose using a recombinant cellulase-displaying yeast strain for high yield ethanol production in consolidated bioprocessing. Bioresour Technol 135:403–409. 10.1016/J.BIORTECH.2012.07.025 22954707 10.1016/j.biortech.2012.07.025

[CR20] Morvan A, Moubareck C, Leclercq A, Hervé-Bazin M, Bremont S, Lecuit M, Courvalin P, Le Monnier A (2010) Antimicrobial resistance of *Listeria **monocytogenes* strains isolated from humans in France. Antimicrob Agents Chemother 54:2728. 10.1128/AAC.01557-09 20385859 10.1128/AAC.01557-09 PMC2876386

[CR21] Murray E, Draper LA, Ross RP, Hill C (2021) The advantages and challenges of using endolysins in a clinical setting. Viruses 13:680. 10.3390/V13040680 33920965 10.3390/v13040680 PMC8071259

[CR22] Nijkamp JF, van den Broek M, Datema E, de Kok S, Bosman L, Luttik MA, Daran-Lapujade P, Vongsangnak W, Nielsen J, Heijne WHM, Klaassen P, Paddon CJ, Platt D, Kötter P, van Ham RC, Reinders MJT, Pronk JT, de Ridder D, Daran JM (2012) De novo sequencing, assembly and analysis of the genome of the laboratory strain *Saccharomyces **cerevisiae* CEN.PK113-7D, a model for modern industrial biotechnology. Microb Cell Fact 11:1–17. 10.1186/1475-2859-11-36/22448915 10.1186/1475-2859-11-36 PMC3364882

[CR23] Nogueira CL, Pires DP, Monteiro R, Santos SB, Carvalho CM (2021) Exploitation of a *Klebsiella* bacteriophage receptor-binding protein as a superior biorecognition molecule. ACS Infect Dis 7:3077–3087. 10.1021/ACSINFECDIS.1C00366/34618422 10.1021/acsinfecdis.1c00366

[CR24] Osek J, Lachtara B, Wieczorek K (2022) *Listeria **monocytogenes* – how this pathogen survives in food-production environments? Front Microbiol 13:866462. 10.3389/FMICB.2022.866462/35558128 10.3389/fmicb.2022.866462 PMC9087598

[CR25] Pottie I, VázquezFernández R, Van de Wiele T, Briers Y (2024) Phage lysins for intestinal microbiome modulation: current challenges and enabling techniques. Gut Microbes 16:2387144. 10.1080/19490976.2024.2387144 39106212 10.1080/19490976.2024.2387144 PMC11305034

[CR26] Ranjbar R, Halaji M (2018) Epidemiology of *Listeria **monocytogenes* prevalence in foods, animals and human origin from Iran: a systematic review and meta-analysis. BMC Public Health 18:1057. 10.1186/S12889-018-5966-8 30139345 10.1186/s12889-018-5966-8 PMC6108140

[CR27] Ribeiro AC, de Almeida FA, Medeiros MM, Miranda BR, Pinto UM, Alves VF (2023) *Listeria **monocytogenes*: an inconvenient hurdle for the dairy industry. Diary 4:316–344. 10.3390/DAIRY4020022

[CR28] Ricci A, Allende A, Bolton D, Chemaly M, Davies R, FernándezEscámez PS, Girones R, Herman L, Koutsoumanis K, Nørrung B, Robertson L, Ru G, Sanaa M, Simmons M, Skandamis P, Snary E, Speybroeck N, Ter Kuile B, Threlfall J, Wahlström H, Takkinen J, Wagner M, Arcella D, Da Silva Felicio MT, Georgiadis M, Messens W, Lindqvist R (2018) *Listeria **monocytogenes* contamination of ready-to-eat foods and the risk for human health in the EU. EFSA J 16(1):e5134. 10.2903/J.EFSA.2018.5134 10.2903/j.efsa.2018.5134 PMC7391409 32760461

[CR29] Schmelcher M, Shabarova T, Eugster MR, Eichenseher F, Tchang VS, Banz M, Loessner MJ (2010) Rapid multiplex detection and differentiation of *Listeria* cells by use of fluorescent phage endolysin cell wall binding domains. Appl Env Microb 76:5745–5756. 10.1128/AEM.00801-10/10.1128/AEM.00801-10 PMC2935047 20622130

[CR30] Tanaka T, Kondo A (2015) Cell-surface display of enzymes by the yeast *Saccharomyces **cerevisiae* for synthetic biology. FEMS Yeast Res 15(1):1–9. 10.1111/1567-1364.12212 25243459 10.1111/1567-1364.12212

[CR31] Turner MS, Waldherr F, Loessner MJ, Giffard PM (2007) Antimicrobial activity of lysostaphin and a *Listeria **monocytogenes* bacteriophage endolysin produced and secreted by lactic acid bacteria. Syst Appl Microb 30:58–67. 10.1016/J.SYAPM.2006.01.013 10.1016/j.syapm.2006.01.013 16490333

[CR32] Välimaa AL, Tilsala-Timisjärvi A, Virtanen E (2015) Rapid detection and identification methods for *Listeria **monocytogenes* in the food chain – a review. Food Control 55:103–114. 10.1016/J.FOODCONT.2015.02.037

[CR33] Yang S, Song L, Wang J, Zhao J, Tang H, Bao X (2024) Engineering *Saccharomyces **cerevisiae* for efficient production of recombinant proteins. Eng Microbiol 4:100122. 10.1016/J.ENGMIC.2023.100122 39628786 10.1016/j.engmic.2023.100122 PMC11611019

