# Supplementary material for: CRISPR-Cas9 engineered Saccharomyces cerevisiae for endolysin delivery to combat Listeria monocytogenes
Source: Appl Microbiol Biotechnol. 2025 Apr 2;109(1):81. doi: 10.1007/s00253-025-13464-8 (PMC11965161; doi:10.1007/s00253-025-13464-8)
Supplement: Supplementary file 1 — Supplementary file1 (PDF 670 kb) [file 253_2025_13464_MOESM1_ESM.pdf]

CRISPR-Cas9 engineered *Saccharomyces cerevisiae* for endolysin delivery to combat *Listeria monocytogenes*

David Sáez Moreno[1,] , Joana Cunha [1,2], Luís Daniel Rodrigues de Melo [1,2,3], Kenya Tanaka[4,5] , Takahiro Bamba[4], Tomosiha Hasunuma[4,5,6], Joana Azeredo[1,2*], Lucília Domingues[1,2*]

[1] CEB - Centre of Biological Engineering, University of Minho, Braga, Portugal.
[2] LABBELS - Associate Laboratory, Braga, Guimarães, Portugal.
[3] Faculty of Pharmacy, University of Coimbra, Coimbra, Portugal
[4] Engineering Biology Research Center, Kobe University, Nada, Kobe, Japan.
[5] Graduate School of Science, Innovation and Technology, Kobe University, Nada, Kobe, Japan.
[6] RIKEN Center for Sustainable Resource Science, 1-7-22 Suehiro-cho, Tsurumi-ku, Yokohama, Kanagawa 230-0045, Japan

[*] Correspondence to: jazeredo@deb.uminho.pt, luciliad@deb.uminho.pt

Supplementary materials:

Table S1. Primers used in this study for plasmid assembly and colony PCR.

Primer name	Primer sequence (5′->3′)	Aim
**p59_FW**	actactttggcccaaGTTAAATACACTGTCGAGAAC	Amplification of the endolysin cassette Ply511-GS-based linker (GSSGGS)-V5tag-GS-based linker(G4S)3-HRV3C cut site
**p54_RV**	taatttactcgagccTGGACCTTGGAATAAGACTT	
**p49_FW**	GGCTCGAGTAAATTATCAAC	Amplification of the plasmid backbone for surface display from pI2-EG-kanMX (*Sed1* promoter, Sed1SS, Sed1 anchor, *Sag1* terminator)
**p56_RV**	TTGGGCCAAAGTAGTC	
**pJ_05_FW**	CCAATTCGCCCTATAGTGAG	Amplification of the insertion site cassette with homology arms for site XI-3, X-4 or XII-5
**pCfB_lin_RV**	ATCGCACGCATTCCATG	
**p_23_9_FW**	tggaatgcgtgcgatttggatatagAAAATTAACGTAAGGCAGTATC	Amplification of surface display of Ply511 cassette
**p_23_10_RV**	tatagggcgaattggTTTGATTATGTTCTTTCTATTTGAATGAGATATGAGAGAG	
**p460_FW**	gctgttatcaaaaag**TAA**TGTACAGTTAGTACATTGAGTC	Amplification of the endolysin secretion without the Sed1 anchor, adding a stop codon (in bold) to close the plasmid.
**p46_RV**	**TTA**CTTTTTGATAACAGCACCCG	
**p62_ply511_FW**	ATTAAGTAAAATCAAGGG	Colony PCR to confirm the insertion of Ply511-GS-based linker (GSSGGS)-V5tag-GS-based linker(G4S)3-HRV3C cut site into the surface display cassette
**pSAG_RV**	GAAGAAAAAAAGAGCCAGGATG	
**p47_FW**	TAAATATACGAAATTATTGTTC	Colony PCR to confirm the insertion of Ply511-surface display cassette into the homology arms for sites XII-5, X-4 and XI-3
**p770_RV**	TTTGCTGGCCTTTTGCTC	

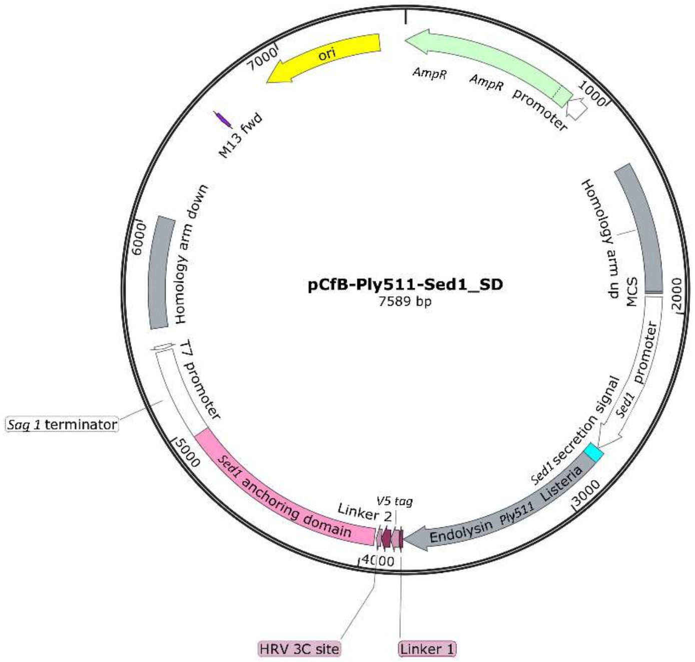

Supplementary Figure S1. pCfB-Ply511-Sed1_SD genome map annotated. Homology regions used in this study belong to X-4, XI-3 and XII-5, sites in pCfB3035-Ply511-Sed1_SD, pCfB2904-Ply511-Sed1_SD, pCfB2909-Ply511-Sed1_SD respectively.

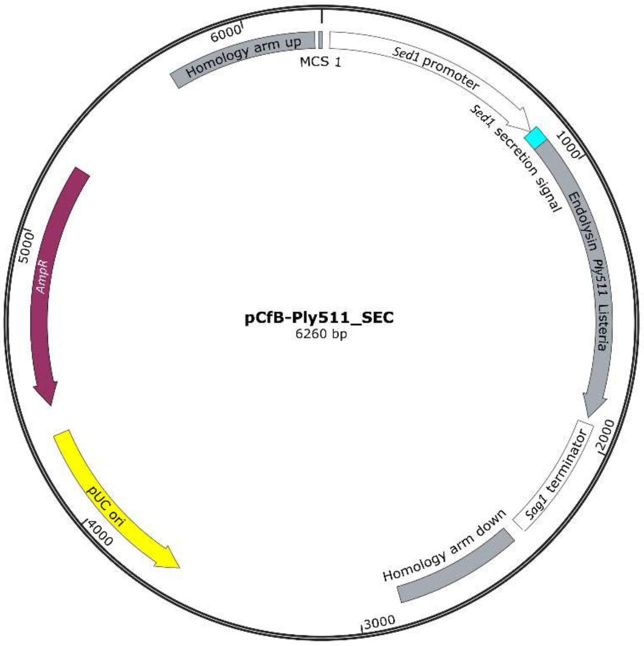

Supplementary Figure S2. pCfB-Ply511_SEC genome map annotated. Homology regions used in this study belong to X-4, XI-3 and XII-5, sites in pCfB3035-Ply511_SEC, pCfB2904-Ply511_SEC, pCfB2909-Ply511-SED1_SEC, respectively.

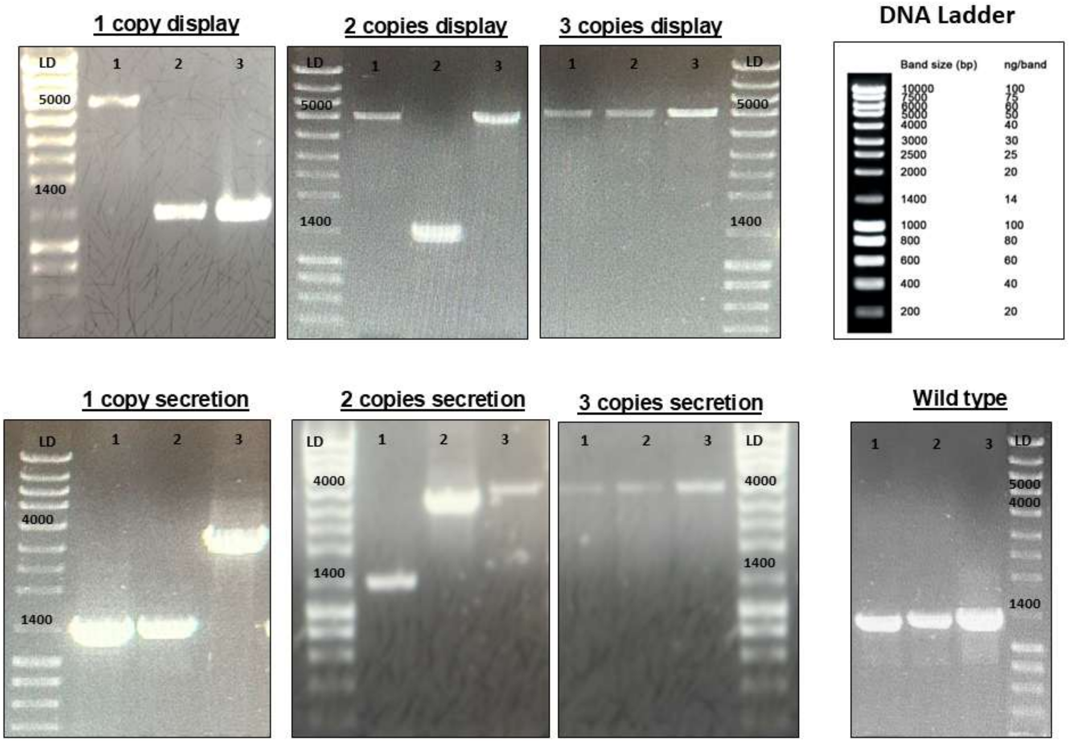

Supplementary Figure S3. Colony PCR of *S. cerevisiae* single, double and triple insertion of the Ply511 display (upper panel) or secretion cassette (lower panel). The picture shows the gel electrophoresis 1% agarose of the PCR products, corresponding to the numbers indicated in the figure legend 1: Chromosome XII site 5 (primers 899 and 900); 2: Chromosome X site 4 (primers 905 and 906) 3: Chromosome XI site 3 (primers 911 and 912), LD: DNA ladder. Sizes expected for unmodified yeast, 1 (XII-5): 1365 bp, 2 (X-4): 1394 bp, 3 (XI-3) 1450 bp. For yeast display, 1 (XII-5): 5128 pb; 2 (X-4): 5157 bp; 3 (XI-3): 5213 bp. For yeast secreting the endolysin, 1 (XII-5): 3799 bp; 2 (X-4): 3828bp; 3 (XI-3): 3884 bp.

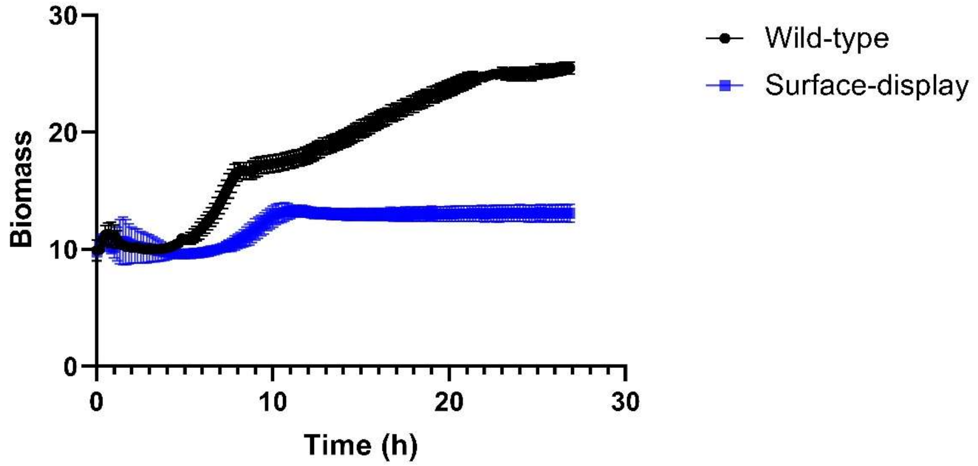

Supplementary Figure S4. Biomass of different yeast over time, as indicated in the legend

Supplementary Table S2. Serovars of *Listeria monocytogenes* used for enzymatic activity confirmation.

**Serovar**	**Strain name**	**Clearance around yeast**
1/2c	CECT 911	Yes
3b	CECT 937	Yes
3c	CECT 938	Yes
4b	Scott A	Yes
4c	CECT 939	Yes

Supplementary Table S3. Values of Log_{10} (CFU/mL) of *L. monocytogenes* Serovar 4c after 3, 6 and 24 h of contact with different yeast concentrations as indicated in the legend. Results are the mean and standard deviation of three replicates.

Yeast 5x10^7	Wild-type	Surface display
3 h	3.12 ± 0.45	3.13 ± 0.11
6 h	4.25 ± 0.10	4.09 ± 0.12
24 h	7.57 ± 0.19	7.58 ± 0.14

Yeast 10^9	Wild-type	Surface display
3 h	3.26 ± 0.02	3.33 ± 0.01
6 h	4.55 ± 0.07	4.29 ± 0.02
24 h	8.6 ± 0.29	8.25 ± 0.13

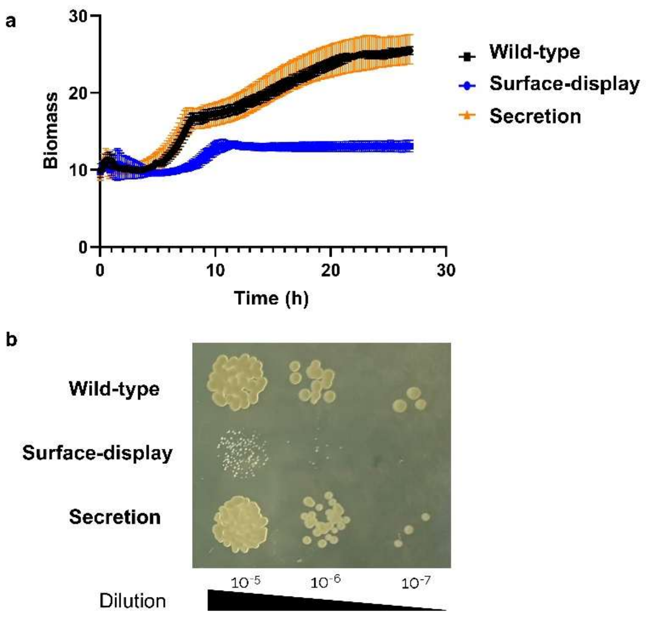

Supplementary Figure S5. a) Biomass over time of different yeast as indicated in the legend. b) Growth of colonies in agar plates after 24 h incubation. (WT: Wild-type yeast, SD: Ply511 Surface-Display yeast; SEC: Ply511 secretion yeast)
